# MARITAL STATUS AND DRINKING BEHAVIORS AMONG OLDER HISPANIC ADULTS IN THE UNITED STATES

**DOI:** 10.1093/geroni/igae098.0492

**Published:** 2024-12-31

**Authors:** Jaminette Nazario-Acevedo, Takashi Yamashita, Jennifer Bulanda, J Scott Brown

**Affiliations:** University of Maryland Baltimore, Baltimore, Maryland, United States; University of Maryland Baltimore County, Baltimore, Maryland, United States; Miami University, Oxford, Ohio, United States; Miami University, Oxford, Ohio, United States

## Abstract

On average, Hispanics are not the most frequent alcohol consumers, however, when Hispanics consume alcohol, the chance of excessive drinking is greater than for other racial and ethnic groups. Excessive drinking can be seen as a stress-coping response and harmful, especially to the health of older Hispanic adults. Hispanic culture places particular importance on family relationships (a.k.a., Familismo). The size of social networks tends to shrink in later life and a specific social relationship-- marriage -- becomes a crucial social support network. Studies show that marital relationships facilitate stress-coping processes and, in turn, benefit health. A social support system can be a resource to prevent stress-induced excessive drinking among older Hispanic adults. Thus, the current study aims to examine how marriage is associated with alcohol consumption behaviors among older Hispanic adults in the United States. The data of the nationally representative samples of Hispanic adults aged 51 years and older (n = 316) are obtained from the 2018 U.S. Health and Retirement Study (HRS). Results from the negative binomial regression indicate that on average, married older Hispanic adults consume 66% fewer alcoholic drinks per week (b = -1.10, p < 0.01) than their unmarried counterparts. Also, older Hispanic women consume 90% fewer alcoholic drinks per week (b = -2.29, p < 0.01) than older Hispanic men. The roles of culturally valued social relationships and gender differences, along with other predictors of alcohol consumption patterns are evaluated from sociocultural and public health perspectives (e.g., prevention of excessive drinking through social support).

